# COVID-19 pandemic in São Paulo: a quantitative study on clinical practice and mental health among medical residency specialties

**DOI:** 10.1590/1516-3180.2021.0109.R1.27042021

**Published:** 2021-06-16

**Authors:** Vitor Silva Mendonça, Amanda Steil, Aécio Flávio Teixeira de Gois

**Affiliations:** I PhD. Psychologist and Research Assistant, Department of Internal Medicine, Faculdade de Medicina, Universidade de São Paulo, São Paulo, SP, Brazil.; II MD. Physician and Medical Resident, Department of Medicine, Universidade Federal de São Paulo (UNIFESP), São Paulo (SP), Brazil.; III MD, PhD. Physician and Professor, Department of Medicine, Universidade Federal de São Paulo (UNIFESP), São Paulo (SP), Brazil.

**Keywords:** COVID-19, Internship and residency, Infant, premature, Mental health, Medical specialties, Coronavirus disease 2019, Mental hygiene, Medical residency

## Abstract

**BACKGROUND:**

2020 was a challenging year for all healthcare professionals worldwide. In São Paulo, Brazil, the virus SARS-CoV-2 took 47,222 lives up to December 29, 2020. The front line of medical professionals in São Paulo was composed of many residents, who were transferred from their rotations to cover the needs of the pandemic.

**OBJECTIVE:**

To identify medical residents’ mental health and clinical issues, regarding symptoms of burnout, depression and anxiety during the pandemic, and to compare them among specialties.

**DESIGN AND SETTING:**

Quantitative study using a convenience sample of medical resident volunteers who responded to an anonymous online survey that was available during April 2020.

**METHODS:**

This investigation collected sociodemographic information and used the Oldenburg Burnout Inventory (OLBI) to measure burnout, the Patient Health Questionnaire (PHQ-9) to measure depression and the General Anxiety Disorder (GAD-7) scale to measure anxiety symptoms. This study also developed a COVID-19 Impact Questionnaire (CIQ-19) to assess the residents’ beliefs and clinical practices relating to COVID-19 patients.

**RESULTS:**

The sample comprised 1,392 medical residents in São Paulo, Brazil. Clinical specialty physicians showed the highest rates of anxiety symptoms (52.6%) and burnout (51.2%), among the specialties.

**CONCLUSION:**

Clinical specialty residents are at higher risk of anxiety, depression and burnout. The symptoms of anxiety and depression have worsened during the COVID-19 pandemic. There is a general need for mental health support interventions for medical resident physicians, which requires reinforcement during this worldwide crisis.

## INTRODUCTION

In December 2019, a new disease caused by the virus SARS-CoV-2, popularly called coronavirus disease 2019 (COVID-19), was identified in Wuhan, China. This disease has high infectivity and transmissibility rates, with a reproductive number greater than one.^1^ Since then, as stated by the World Health Organization (WHO), as of December 29, 2020, there were more than 79,231,893 cases and 1,754,574 deaths worldwide. Brazil was the epicenter of the pandemic with more than 7,448,560 cases and 190,488 deaths. São Paulo had 1,486,551 cases and 47,222 deaths up to December 29, 2020.^2^

Around the world, physicians of various specialties were called up to work in the fight against COVID-19.^3^^,^^4^^,^^5^^,^^6^ Among these professionals, medical residents were reallocated from their rotations, to work in emergency departments, intensive care units and COVID-19 wards, in order to supply the need for medical personnel.^7^,^8^

São Paulo is the most populous city in Brazil and also the fourth largest city in the world by population. This city is an important focal point for the field of healthcare and has the highest concentration of physicians in the country, around 146,970 professionals. Among them, 18,236 were working as medical residents in 2019, which represented 33.9% of all medical residents in Brazil.^9^

Along with training and structural safety issues, the psychological effects on healthcare workers need to be discussed. During the last decade, motivated by alarming numbers of cases of mental illness and suicides in the medical community, the issues of mental health and the need for new perspectives of care have been highlighted.^10^^,^^11^^,^^12^ Previous studies that analyzed the psychological effects of emerging virus outbreaks on healthcare workers found that staff in high-risk areas exhibited increased levels of acute or posttraumatic stress and psychological distress.^13^ The risk factors included younger age and less experience: two aspects that may be related to medical residents, a group that clearly needs guidance from more experienced staff to help them achieve the required knowledge and ethical maturity to deal with the difficulties and feelings inherent to this period, which can be an extremely rich learning experience.^14^

## OBJECTIVE

The purpose of this study was to identify the mental health and clinical issues of medical residents in São Paulo, Brazil, regarding collective symptoms of burnout, depression and anxiety during the COVID-19 pandemic and compare them among medical specialties.

## METHODS

### Study design and procedures

A quantitative study was conducted in accordance with the Strengthening the Reporting of Observational Studies in Epidemiology (STROBE) parameters, to identify the clinical practice and mental health issues of medical residents in São Paulo, regarding their collective symptoms of burnout, depression and anxiety during the COVID-19 pandemic.

This study used a convenience sample from an anonymous online survey that was advertised through social media and distributed by means of e-mails from the residency committees of hospitals that are linked to universities and medical associations in São Paulo. Given that a convenience sample was used, no calculation of sample size was performed. Advertisement of the research was performed in line with the good practice guidelines.^15^ There was no financial support for the volunteers who responded to the survey. The survey was available during the month of April 2020 in São Paulo.

### Study measurements and analysis

This investigation collected sociodemographic information and used the Oldenburg Burnout Inventory (OLBI) to measure burnout, the Patient Health Questionnaire (PHQ-9) to measure depression and the General Anxiety Disorders (GAD-7) to measure anxiety symptoms.^16^^,^^17^^,^^18^ All three scales had previously been adapted and validated for use in Brazilian contexts and populations. This study also developed a COVID-19 Impact Questionnaire (­CIQ-19) to assess the residents’ beliefs and clinical practices relating to COVID-19 patients, their behaviors concerning disease prevention and their substance use after the beginning of the pandemic. All fields were marked as mandatory, so a participant could move forward only after answering all questions. Therefore, all the participants included completed the entire questionnaire, and no data were missing. The protocol was reviewed and approved by the Universidade Federal de São Paulo (UNIFESP) Research Ethics Committee (Protocol #3,943,348; on March 20, 2020).

Exploratory analyses were conducted using basic contingency tables with analysis of variance (ANOVA), Mann-Whitney U test and Fisher’s test. All analyses were controlled according to specialty areas: clinical specialties, surgical specialties and diagnostic and therapeutic support (i.e. pathology, radiotherapy and nuclear medicine). The residents’ sociodemographic variables, gender and ethnicity characteristics, the nature of the hospital (public or private), year of medical residency and contact with ­COVID-19 patients were described. We also identified clinical issues and beliefs regarding COVID-19 and mental healthcare. Burnout was defined as positive if the total score on OLBI was 21; anxiety was defined as positive if the total score on GAD-7 was 10 or greater. For the variable “depression”. three categories were used: no depression or mild depression (PHQ-9 score of nine or less), moderate (PHQ-9 score between 10 and 14) and severe depression (PHQ-9 score of 15 or higher).

The analyses were performed using SPSS Statistics for Windows, version 22.0 (released 2013; IBM Corp, Armonk, NY, United States) and the statistical significance level was taken to be 0.05. The results were presented as proportions and distributions of scores in the categories of each scale (frequencies).

## RESULTS

This sample comprised 1392 residents in São Paulo, Brazil. The response rate represented approximately 8% of the medical residents in the state. These residents comprised 914 in clinical specialties, 336 in surgical specialties and 142 in diagnostic and therapeutic support. Most were women (72.5%), white (81.0%), first-year residents (35.4%), in a program provided by a public hospital (84.8%) and in close contact with COVID-19 patients (69.8%). The mean age of the sample was 27.9 years (standard deviation, SD: 3.0) (Table 1).

**Table 1.** Descriptive statistics on medical residents in Sao Paulo, 2020

		Specialty of medical residency^a^								
Descriptive		Clinical specialties n = 914		Surgical specialties n = 336		Diagnostic and therapeutic support n = 142		Total n = 1392		P-value
		n	%	n	%	n	%	n	%	

**Gender**	Male	202	22.1	125	37.2	55	38.7	382	27.4	< 0.05
	Female	702	76.8	211	62.7	87	61.2	1010	72.5	
**Ethnicity**	White	746	81.6	271	80.6	111	78.1	1128	81.0	> 0.05
	Non-white	168	18.3	65	19.3	31	21.8	264	18.9	
**Nature of hospital**	Public	807	88.2	285	84.8	89	62.6	1181	84.8	< 0.05
	Private	107	11.7	51	15.1	53	37.3	211	15.1	
**Year of residency**	R1	325	35.5	119	35.4	49	34.5	493	35.4	< 0.05
	R2	269	29.4	95	28.2	43	30.2	407	29.2	
	R3	163	17.8	66	19.6	44	31.0	273	19.6	
	R4	122	13.3	27	8.0	4	2.8	153	11.0	
	R5	30	3.2	21	6.2	2	1.4	53	3.8	
	R6	5	0.5	8	2.3	-	-	13	0.9	
**Do you have contact with COVID-19 patients?**	Yes	674	73.7	201	59.8	98	69.0	973	69.8	< 0.05
	No	240	26.2	135	40.1	44	31.0	419	30.1	

^a^As described in the Methods section.

Our findings were significant with regard to mental health scales, COVID-19 aspects of clinical practice and mental healthcare among medical residents training in São Paulo.

Depressive symptoms were the most common (65.8%), followed by anxiety symptoms (49.7%) and burnout (49.2%) among these residents. We also observed anxiety symptoms (52.6%) and burnout (51.2%) among clinical specialties (Table 2).

**Table 2.** Mental health scale scores for medical residents in São Paulo, 2020

		Specialty of medical residency								
Mental Health		Clinical specialties n = 914		Surgical specialties n = 336		Diagnostic and therapeutic support n = 142		Total n = 1392		P-value
		n	%	n	%	n	%	n	%	

**Depression^a^**	Absent or mild	287	31.4	137	40.7	51	35.9	475	34.1	< 0.05
	Moderate	244	26.6	93	27.6	46	32.3	383	27.5	
	Severe	383	41.9	106	31.5	45	31.6	534	38.3	
**Anxiety^b^**	Absent or mild	433	47.3	191	56.8	76	53.5	700	50.2	< 0.05
	Moderate or severe	481	52.6	145	43.1	66	46.4	692	49.7	
**Burnout^c^**	Absent or mild	446	48.7	182	54.1	78	54.9	706	50.7	< 0.05
	Moderate or severe	468	51.2	154	45.8	64	45.0	686	49.2	

^a^According to the Patient Health Questionnaire (PHQ-9); ^b^According to the General Anxiety Disorder (GAD-7) scale; ^c^According to the Oldenburg Burnout Inventory (OLBI).

In terms of aspects of COVID-19, residents in surgical specialties were less likely to feel that they themselves and their hospital were sufficiently prepared to treat patients with this disease (46.4% and 33.6%, respectively). The residents in these specialties also revealed that there was a lack of supervisor support for treating COVID-19 patients (55.6%). 

Residents said that their personal protection equipment (PPE) was not efficacious (47.2%), felt that they would be better professionals after the pandemic (80.3%), felt significantly worried about getting COVID-19 and transmitting it to their partners and families (90.5%) and had experienced impairment of personal relationships since the pandemic started (75.0%). There was no significant increase in substance use among them (Table 3).

**Table 3.** Frequencies of perceptions regarding COVID-19 among medical residents in São Paulo, 2020

		Specialty of medical residency^a^								
		Clinical specialties n = 914		Surgical specialties n = 336		Diagnostic and therapeutic support n = 142		Total n = 1392		P-value
		n	%	n	%	n	%	n	%	
**COVID-19 clinical practice and beliefs**	The hospital is prepared for treating patients with COVID-19	704	77.0	156	46.4	99	69.7	959	68.8	< 0.05
	Residents feel prepared for treating patients with COVID-19	453	49.5	113	33.6	92	64.7	658	47.2	< 0.05
	Supervisors do not provide the necessary support for treating patients with COVID-19	203	22.2	187	55.6	77	54.2	467	33.5	> 0.05
	Hospital provides personal protection equipment	449	49.1	120	35.7	88	61.9	657	47.2	< 0.05
	I feel I will be a better professional after the pandemic	744	81.4	269	80.0	106	74.6	1119	80.3	< 0.05
**COVID-19 behaviors**	Afraid of getting COVID-19 and transmitting it to my partner and family	841	92.0	303	90.1	117	82.3	1261	90.5	> 0.05
	Personal relationships impaired since the pandemic started	737	80.6	205	61.0	102	71.8	1044	75.0	< 0.05
**Substance use after pandemic started**	Increase in alcohol use	325	35.5	39	11.6	56	39.4	420	30.1	> 0.05
	Increase in tobacco use	350	38.2	71	21.1	42	29.5	463	33.2	< 0.05

Our sample revealed how the medical residents had been coping with their mental health. Residents in clinical and surgical specialties preferred to talk with family or friends (48.8% and 29.0%, respectively). Those in surgical specialties also chose to discuss matters with their team support (26.3%) when they need mental healthcare. On the other hand, this same group did nothing about their mental healthcare (17.8%), and only 4.0% among the residents in clinical specialties mentioned psychotherapy in relation to their mental healthcare (Table 4). 

**Table 4.** Frequency of mental healthcare among medical residents in São Paulo, 2020

		**Specialty of medical residency**							
		Clinical specialties n = 914		Surgical specialties n = 336		Diagnostic and therapeutic support n = 142		Total n = 1392		P-value
		n	%	n	%	n	%	n	%	
**Mental healthcare^a^**	Talking to friends/family	758	48.8	194	29.0	69	13.5	1021	37.4
	Team support	302	19.4	176	26.3	84	16.5	562	20.6
	Physical activity	264	17.0	46	6.8	97	19.0	407	14.9	< 0.05
	Nothing	111	7.1	119	17.8	69	13.5	299	10.9	
	Others	54	3.4	76	11.3	99	19.4	229	8.3	
	Psychotherapy	63	4.0	57	8.5	90	17.7	210	7.6	
**Total responses**		**1552**	**56.8**	**668**	**24.4**	**508**	**18.6**	**2728**	**100.0**		

^a^Multiple responses.

## DISCUSSION

Three main findings emerged from this study among medical residents from a convenience sample in São Paulo: depression, anxiety and burnout are frequent among residents in clinical specialties working in the COVID-19 pandemic; exposure to COVID-19 patients may be related to changes in clinical practice, personal behavior and substance use in all three specialty areas; different fears, confidences and beliefs showed that there was low mental healthcare in the three specialty areas.

Medical residents’ mental health was already a topic that worried medical educators worldwide, even before the pandemic scenario emerged. In the light of this worldwide scenario, we observed that clinical specialties had higher rates of depression, anxiety and burnout symptoms than other ones. A previous study reported that the prevalence of depression or depressive symptoms among resident physicians was 28.8%, with a range from 20.9% to 43.2%.^11^ In 2014, the prevalence rates for anxiety, depression and burnout were 41.3%, 21.6% and 58.4% respectively, among Brazilian residents. Thus, although the prevalence of burnout that was identified was similar to that of the pre-pandemic scenario, symptoms of anxiety were increased twofold and symptoms of depression exhibited an approximately threefold increase in the scenario of the COVID-19 pandemic.^19^ These findings are similar to those of a survey of 1,257 healthcare workers who were in contact with COVID-19 patients in China, in which high rates of depression (50.4%), anxiety (44.6%) and distress (71.5%) were reported using the same instruments as those used in our study.^20^ Mental distress was also reported in Italy, where only 20% to 25% of healthcare workers declared that they felt psychologically safe and only 48% reported having access to mental healthcare.^21^

Another study observed that the prevalence of burnout among surgical residents was 69%. This rate was associated with high levels of stress, depression and suicidal ideation. Surgical trainees have purposefully chosen a field with high baseline stress by definition, presumably due in part to the widely held belief among surgeons that not all stress is bad. Although burnout among them is known to be high, it is not clear whether stress and distress have the same strong associations with burnout, depression and anxiety symptoms, as seen in other medical specialties.^22^

Unfortunately, we did not find any Brazilian studies that compared medical specialties. To our knowledge, our study is the first to show a relevant difference among residents in medical specialties regarding the collective symptoms of burnout, depression and anxiety. Similar results have been found recently in studies among medical residents without comparing areas of specialties.^11^,^19^ Two of these studies included Brazilian residents as participants. Given our findings relating to different behaviors among medical residents exposed to patients infected with or suspected of being infected with COVID-19, and the factors correlated with the development of mental disorder symptoms, it is necessary to develop interventional programs to prevent mental illness.

This investigation found that residents did not feel prepared to treat patients with COVID-19 and were afraid of getting it. Since these individuals believed that talking to colleagues and immediate line managers protected their mental health, it can be suggested that supervisors and peers should pay attention to residents with signs of at-risk behavior.^23^ However, in the surgical specialty, the residents stated that there was a lack of support from supervisors during the treatment of these patients. Resident support groups can be a powerful resource for preventing psychological impacts, along with wider support networks, including the friends and family of medical residents, who may help them by keeping in touch through online resources. However, 10.9% of the residents stated that they had not had any care for their mental health during the COVID-19 pandemic.

There are only a few studies on medical residents’ mental health during the COVID-19 pandemic. Although residents represent only a fraction of healthcare workers, their participation on the COVID-19 frontline is evident. A previous study highlighted that healthcare professionals suffer more stigma than the general population and are consistently more affected psychologically after quarantine.^24^ Our participants reported that their personal relationships had become impaired since the pandemic started. This situation may lead to a greater chance of developing burnout, anxiety and depression symptoms.

A review on Brazilian medical residents indicated that in the state of São Paulo, most physicians are concentrated in the clinical specialty, in which supervisors are perceived to be important in the clinical practice process. That study also corroborates our findings, through stating that significant situations such as a pandemic or an outbreak of a certain disease make the medical professional realize the importance of residents’ work and favors their professional growth. In our study, 80.3% of residents perceived this, in affirming they may become better professionals through their experience in the pandemic.^9^

The results from the present study suggested that there had not been any significant increase in substance use among these residents. However, another study found that residents in surgical specialties were more likely to present alcohol dependence than were residents in clinical specialties. Another study among practicing surgeons in the United States showed that alcohol abuse and dependence are a significant problem in that population. It is necessary to assess the relationship between substance dependence and personality among medical residents, since these are risk factors for this population.^25^,^26^

The cross-sectional design chosen for the present study was suitable for investigating associations and for providing wide-ranging data for discussion, but it does not allow inferences of causality. In addition, since our sample was restricted to one state in Brazil, further studies should investigate whether the trend cited above is replicable in other countries. Therefore, caution is needed with regard to generalizations of these results to distinct populations.

## CONCLUSIONS

This study was conducted during the worldwide crisis of the COVID-19 pandemic. It is understandable that most of our sample of physicians undergoing training felt unprepared to deal with COVID-19 patients. This was one of the variables related to increased mental illness, such as depression, anxiety and burnout.

Residents in clinical specialties seem to be at higher risk of depression, anxiety and burnout during the pandemic, but previous studies that have been conducted outside of this scenario have shown the same results. Our findings from comparisons between specialties among Brazilian resident physicians highlights the need to better explore mental health in this population, since surgical specialties did not follow a trend towards higher rates, which was restricted to the clinical group.

This study demonstrates that there is a general need for better access to mental health professionals for resident physicians, which has been reinforced during the COVID-19 pandemic. Moreover, the feeling that support from staff is available appears to have a protective function in relation to mental health. Therefore, we suggest that support groups might give rise to the possibility of assessing mental health. We also emphasize that there is a need for more comparative studies between medical specialties.
